# Novel Approaches to Ileocolic and Perianal Fistulising Crohn's Disease

**DOI:** 10.1155/2018/3159543

**Published:** 2018-11-21

**Authors:** Gaetano Luglio, Gianluca Cassese, Alfonso Amendola, Antonio Rispo, Francesco Maione, Giovanni Domenico De Palma

**Affiliations:** ^1^Department of Clinical Medicine and Surgery, University of Naples Federico II, Italy; ^2^Center of Excellence for Technical Innovation in Surgery, Italy

## Abstract

Crohn's disease (CD) is a well-known idiopathic inflammatory bowel disease characterised by transmural inflammation which can ordinarily affect all the gastrointestinal tract. Its true aetiology is unknown, and a causal therapy is not available to date. The most peculiar aspect of CD lies in its absolute heterogeneity, as we might face various scenarios, locations of the disease, pathologic behaviours, and severity of the disease itself. For these reasons, the cornerstone for the treatment of CD lies in a complex multimodal management, requiring close collaborations among surgeons, gastroenterologists, radiologists, and staff nurses. Advances in surgical and medical therapy are changing the course of the disease. Nowadays, the introduction of both laparoscopy and novel surgical techniques, the improvement of recovery pathways, and the opening of new frontiers are allowing healthcare professionals to deal with complex and recurrent scenarios, trying to spare bowel and anal function, thus ensuring a better quality of life for the patient. Given the heterogeneity and complexity of this disease, it would be impractical to encompass all the aspects of surgical management of CD. This review will address areas that are considered to be hot topics, controversies, challenges, and novelties: thus, we will focus on complex ileocecal disease, surgical strategies, and fistulising perianal conditions.

## 1. Introduction

Crohn's disease (CD) is a well-known idiopathic inflammatory bowel disease characterised by transmural inflammation, which can ideally affect all the gastrointestinal tracts, from the mouth to the anus. It is a lifelong disease whose pathogenesis is unknown, depending on the interaction of both genetic and environmental factors, mainly observed in developed and Western countries. As its true aetiology is not fully understood, a causal therapy is not available to date, and CD often requires a complex multimodal management, based on the close collaborations among gastroenterologists, surgeons, and radiologists, at least. The most peculiar aspect of CD probably lies in its absolute heterogeneity, as we might face lots of different clinical scenarios, different locations of the disease, different pathologic behaviours, and different levels of severity [1]. From a clinical perspective, it is mainly characterised by abdominal pain, diarrhoea, weight loss, and systemic involvement such as fever, anorexia, or extraintestinal manifestation. As mentioned above, the location of the disease can vary: the Montreal classification identifies ileal, colonic, and ileocolic diseases, which tend to remain stable over time [2, 3], while its phenotypes (inflammatory, stricturing, and penetrating) tend to change over time, along with disease severity [4]. Fistulising perianal Crohn's disease is another clinical entity: in population-based studies [5–7], the occurrence of perianal fistulas in CD varies between 14 and 23% of cases, with cumulative incidence depending on time from diagnosis. This latter condition is of much interest because it is associated with a more aggressive disease course and the management has quite dramatically changed in recent years, requiring a strong collaboration between surgeons and gastroenterologists.

The standard treatment of CD is based on both medical and surgical options; medical treatments include steroids, immunosuppressants (e.g., azathioprine) and, in recent years, biologic agents. The best-known biologics are infliximab and adalimumab, anti-TNF monoclonal antibodies, which have demonstrated the ability to induce and maintain remission [8, 9]. Their most frequent use is seen in severe and aggressive CD, refractory to other treatments. On the other hand, the idea of a “top-down” approach strategy, based on the early introduction of combined immunosuppressants and biologics since the first flare of disease in “high-risk” patients, is currently widely debated and is considered the theoretical benefit of changing disease course, avoiding the progression of bowel damage [10].

Despite significant advances in medical therapies, a high percentage of patients with CD will require surgery during their lifetime for a primary or recurrent disease. A meta-analysis of 30 population-based studies showed that the cumulative risk of surgery in CD is 16% at one year after the diagnosis and up to 47% at 10 years [11]. Surgery is usually required to deal with disease-related complications or refractoriness to medical treatment; more, another crucial issue is the recurrent disease, considering that up to 40–50% of patients will require repeated surgery in 10–15 years' time after the first operation [12], creating further challenges for both surgeons and gastroenterologists.

Given the heterogeneity and complexity of this disease, it would be impractical to encompass all the aspects of surgical management of CD. This review paper will address areas that are considered to be hot topics, controversies, challenges, and novelties: thus, we will focus on complex ileocecal disease, laparoscopy and “ERAS” (enhanced recovery programme after surgery), surgical strategies to possibly prevent recurrences, and complex fistulising perianal conditions.

## 2. Ileocecal Crohn's Disease

The dramatic improvements in medical therapy somehow put the primary management of Crohn's disease in the hands of gastroenterologists, who can face situations that could only be treated surgically some years ago. From this perspective, surgery could be considered a kind of last resort, mainly used to treat complicated diseases. By contrast, long-term studies have demonstrated that there is a 50% chance of not requiring any further operation for CD [13, 14] after primary ileocecal resection. The idea of an early surgery has been debated for a long time [15–17]: the issue is whether to consider surgery to be the “end of the road”, useful when any other medical options have failed, a part of a multimodal strategy, or even an alternative to long-term medical therapy. This possibility is even more appealing, considering that surgery for primary disease is usually technically easier and has lower complication rates compared with surgery for recurrent disease and after immunosuppressant therapy. The main problem in implementing early surgery in clinical practice has been the lack of prospective and randomised data, comparing results, even in terms of quality of life, after surgery and chronic medical therapy. From this standpoint, results from the LIR!C trial study group in 2017 have provided crucial insights and will be further discussed below.

There are very few randomised trials driving the decision-making in the surgical management of CD; nevertheless, a multimatrix model was suggested to stratify the risk for patients undergoing surgery within one year, based on the combination of endoscopic and ultrasonographic findings [18]. On the other hand, there is strong evidence to support the principle that extensive resection is not necessary [19] and is potentially harmful in the surgical management of Crohn's disease. In other words, surgery in CD should be aimed at treating the segment of the bowel causing symptoms or responsible for complications [20]. There is no need to get wide “free margins” or perform extensive resection if the disease can be treated medically. In fact, extensive and repeated surgery is potentially harmful because of the risk of short bowel.

The latest available ECCO (European Crohn's and Colitis Organization) guidelines assess that stricturing localised ileocecal CD with obstructing symptoms and not significant active inflammation needs to be managed surgically [21]. When inflammatory activity is present, the bowel affected is no longer than 40 cm. With no symptoms of imminent obstruction, steroid treatment could be taken into account. Certainly, surgery is the first option for patients with persistent obstructive symptoms, refractory to the initial steroid treatment. The same is probably true for people with obstructive symptoms and no signs of active inflammations evaluated, for example, CRP. In these cases, an anti-inflammatory therapy with steroids is useless, and surgery in fibrotic stenosis is certainly the best option.

Fistulising ileocecal CD, especially if complicated by a concomitant abdominal abscess, might be another challenging situation. When a clear abscess is present, the first option could be medical therapy based on antibiotics and, eventually, a percutaneous drainage [21]. In these cases, resection is possibly postponed to avoid the risk of an anastomosis in a septic environment and the subsequent risk of a stoma. A delayed elective resection is usually necessary even though evidence from the literature regarding elective resection after the drainage of an abscess is not strong [22, 23]. Zerbib et al. [24] also emphasised the importance of appropriate preoperative management and patient optimisation: drainage of abscesses, bowel rest, nutritional therapies, IV antibiotics, weaning off steroids, and immunosuppressants lead to low rates of both postoperative morbidities and faecal diversion. They retrospectively collected data from 78 consecutive patients undergoing primary ileocecal resection (from 1997 to 2007) after proper preoperative optimisation, reporting 18% of overall postoperative morbidity, with only 5% of major complications. A low temporary stoma of 7.7% was also recorded, mainly in patients either with residual abscess or with ileosigmoidal fistulas, necessitating a wide sigmoidal resection.

With regard to the effect of medical therapy on surgical outcomes, there is a consensus and good evidence that surgery under steroids (20 mg of prednisolone or equivalent) is impaired by a higher rate of septic and wound complications and should thus be weaned when possible [25, 26]. From this standpoint, another hot topic is represented by the role of biological therapy in influencing surgical outcomes and complications.

The risk of a higher postoperative complication rate after anti-TNF therapy is still controversial, and a clear safety interval between the last administration and surgery has not been determined yet. The literature is quite contradictory, as there are reports not supporting the evidence for higher complication rates after biologics [27, 28] and other studies warning a significantly higher risk of postoperative septic complications [29, 30]. This has also been demonstrated both in a meta-analysis [31] and in a paper focusing on outcomes after ileocecal resection within three months from the last administration of infliximab, showing a higher readmission and postoperative septic complication rate [32].

In a recent large nationwide prospective cohort study by Broquet et al. [30], including 592 patients from 19 French academic centres from the GETAID Chirurgie group, the authors demonstrated that the use of an anti-TNF drug within 3 months prior to surgery is associated with increased overall postoperative morbidity at both univariate and multivariate analyses. More, as the use of a biological drug is a confounding factor, being associated with the severity of the disease, a propensity score was also calculated, considering the risk factors found at multivariate analysis, again demonstrating that the anti-TNF therapy 3 months prior to surgery increases the risk of both overall postoperative morbidity and intra-abdominal septic complications.

## 3. Recurrences and Surgical Issues

The recurrence risk challenges surgeons and gastroenterologists who are involved in CD treatment. Postoperative endoscopy follow-up data show that in the absence of medical treatment, the rate of endoscopic recurrence can reach 80–100% in 3 years after surgery; the clinical recurrence rate is instead 20–25%/year [33].

In a recent study, Auzolle et al. [34] have investigated several factors as predictors of early postoperative recurrence after ileocecal resection for CD. They concluded that male gender (OR = 2.48), being an active smoker at the time of surgery (OR = 2.65), and previous surgery (OR = 3.03) were independent factors associated with a higher risk of postoperative recurrence. Being a patient-related modifiable factor, smoking habit appears to be the most important risk factor for recurrence in CD. Recently, Nunes et al. [35] also demonstrated that smoking cessation could improve CD prognosis. In their four-year follow-up study, the authors found out that patients quitting smoking had a similar prognosis to that of nonsmokers, while those who continued smoking were over 50% more likely to recur during the follow-up period.

On the other hand, the role of surgery-related factors in avoiding recurrence has been poorly investigated. Despite significant advances in medical therapy, most relapses still appear at the anastomotic site which underlines that surgery itself plays some causal role. A different ileocolic anastomotic configuration has been described, but so far no clear advantage has been demonstrated in terms of relapse prevention. ECCO guidelines [21] support the use of a stapled side-to-side anastomosis after ileocolic resection as the technique of choice. Most relapses appear only proximally to the anastomosis, which led to thinking that the anastomotic configuration and subsequent faecal stasis could play a role. On the other hand, a side-to-side stapling anastomosis never demonstrated any advantage in preventing recurrences, and the recommendation is then based on the results of two meta-analyses showing an advantage of the side-to-side anastomosis in reducing the anastomosis leak over the end-to-end anastomosis [36, 37]. Other studies, however, did not reach the same conclusion [38].

A novel anastomotic configuration has been described by Kono et al. in 2011 [39], combining stapled and hand-sewn antimesenteric functional end-to-end anastomosis (Kono-S anastomosis) to reduce surgical recurrence. From a technical point of view, the anastomosis is performed, cutting the ileal and the colonic edge with a linear cutter, locating the mesentery at the centre of the stump, perpendicularly to the staple line. The bowel needs to be cut really close to the bowel wall to minimise any devascularisation or denervation. The two staple lines are then approximated with interrupted stitches to create a kind of supporting column to prevent any further anastomotic distortion. The anastomosis itself is then created, performing two longitudinal enterotomies, 7 cm long, at the antimesenteric side, which are then reapproximated in one or two layers in a transverse fashion.

As previously mentioned, the anastomotic configuration is supposed to be responsible for faecal stasis, bacterial overgrowth, and bowel perfusion. A wide-lumen stapled side-to-side anastomosis was thought to reduce faecal stasis, eventually reducing the recurrence risk; however, it has never been demonstrated.

In their another study, Kono et al. [39] report excellent results in a group of 69 patients who underwent a novel Kono-S anastomosis between 2003 and 2009 when compared with the historical group of 73 patients receiving a conventional anastomosis.

They found that the median endoscopic recurrence score was significantly lower in the Kono-S group, resulting in a reduced risk of surgical relapses (0% vs. 15%, *P* < 0.0013). The mechanism of the anastomosis in reducing the risk of recurrence deserves some attention from our point of view:

1. The anastomosis itself, being constructed in a transverse fashion, similar to a strictureplasty, creates a large lumen
2. The staple lines, on the back of the anastomosis, create a supporting column able to prevent anastomotic distortion in case of recurrence, so that the risk of stenosis associated with recurrent disease is lower
3. More interestingly, the possibility of excluding the mesentery from the anastomosis lumen exists

It has been shown that the CD always appears and recurs on the mesenteric side [40], which is hidden on the back of the Kono-S anastomosis in the centre of the support column. Further advantages of this anastomosis are the maximum preservation of blood supply and innervation, which should be both factors associated with CD recurrence.

A broader multicenter series including an institution from the United States has been recently published with the same interesting results [41].

A randomised controlled trial is currently underway at our institution; it was registered on ClinicalTrials.gov (NCT02631967) and compares the Kono-S anastomosis with the standard side-to-side anastomosis. Regardless of the encouraging result obtained in a patient with a severe multirecurrent CD, not relapsing after Kono-S anastomosis to date [42], the preliminary results on our series are very promising and have been presented at the last ECCO meeting in Amsterdam [43].

With regard to the role of surgery in influencing the natural history of CD, a recent study by Coffey et al. [44] claimed, for the first time, that the inclusion of the mesentery as part of intestinal resection is associated with reduced surgical recurrence. In fact, even though it is usually believed that the mesentery can be left in place after bowel resection for CD, the authors compared surgical recurrence in two cohorts. In cohort A (30 patients), a standard ileocecal resection was performed, cutting the mesentery close to the bowel wall. In cohort B (34 patients), the mesentery supplying the ileocecal diseased segment was instead fully mobilised and resected together with the bowel. Surprisingly, the cumulative reoperation rate in cohort A was 40%, while it was only 2.9% in cohort B. Most of the recurrences happened within two years after surgery. Mesentery-sparing resection was, therefore, recognised as an independent risk factor for surgical recurrence. Coffey et al. also developed a novel “mesenteric disease activity index” based on the presence of “fat wrapping” and “mesenteric thickening”: other than being related to the Crohn's Disease Activity Index (CDAI) and to the percentage of circulating fibrocytes, it was also found to predict surgical recurrence.

Strictureplasties have proven to be a valid and safe alternative to resection surgery in the jejunal-ileal CD. Heineke-Mikulicz strictureplasty [45] is the most commonly performed and is generally referred to as the “conventional strictureplasty.” It is based on a longitudinal enterotomy on the antimesenteric side of the stenotic bowel and a subsequent transverse closure.

The conventional strictureplasty is generally recommended for stenosis no longer than 10 cm [21]. A strictureplasty is usually indicated in the case of an isolated stricture, far from a resection site. They have a prominent role, especially in plurioperated patients, to avoid additional resections and the risk of short bowel. Strictureplasties are easier to perform and particularly indicated in cases of long-standing and fibrotic stenosis. On the other hand, if the stenosis has an active inflammatory component not suitable for medical therapy, care must be taken during surgery due to the likelihood of intra- and postoperative bleeding. Strictureplasties have some contraindications: the presence of a free or contained bowel perforation, the presence of multiple stenosis in a short-bowel segment (where a single resection is preferred instead), a stenosis close to a resection site (it is better to include the stenosis in a single resection), hypoalbuminemia (<2 gr/dl), and the presence of adenomesenteritis (it is difficult and tricky to approximate the two margins of the intestine to perform closure in these cases).

“Unconventional” strictureplasties for longer strictures have been described too, and both their safety and efficacy have been proven. Finney-type [46] (side-to-side antiperistaltic) and Michelassi [47] (side-to-side isoperistaltic) strictureplasties have been widely used for strictures longer than 10 cm, with very good results [48–50]. A multicentre prospective study with Michelassi-type strictureplasty for long strictures (20–100 cm) has shown positive results in terms of feasibility, postoperative complications, and surgical relapses [51]. Interestingly, it has been observed that CD appears to have a very low relapse rate at the strictureplasty site compared to anastomosis [52, 53]; on the other hand, despite the fact that very few cases have been reported from literature, the potential risk of cancer at the strictureplasty site should be borne in mind [54].

## 4. Laparoscopy and Enhanced Recovery Programme

Laparoscopy is now considered the preferred surgical option to treat ileocecal CD when appropriate expertise is available [21]; on the other hand, the evidence is consistently less strong when we face a complex or recurrent disease.

Laparoscopic surgery has been demonstrated to be safe and feasible in treating both benign and malignant colorectal diseases, even when performed by trainees, making it possible to achieve benefits in terms of cosmetics, postoperative pain, restoration of bowel function, and shorter recovery and length of hospital stay without compromising other surgical outcomes [55–58].

Similar results, showing the benefits of the laparoscopic approach, were demonstrated by several studies focused on outcomes after laparoscopic resection for ileocecal Crohn's disease, without significant differences in terms of recurrences [59–61]. A trend in favour of reduced postoperative complications, including wound infections, incisional hernias, and adhesion formation, has also been reported [62], showing potential benefits even in the setting of recurrent disease. On the other hand, the big issue lies in the extreme heterogeneity of CD: we can face an isolated, localised, stricturing ileocecal disease, really easy to treat laparoscopically, or a complex fistulising disease, with thick adenomesenteritis or fistulas with other bowel loops, sigmoid, bladder, and ureter; more, the clinical scenario could be even worse in case of recurrent or multirecurrent disease. The presence of abscesses and fistulas, together with disease severity itself, seems to be the most important factor associated with conversion [63], leading to increased operative time and cost and slightly higher postoperative complications [64]. We should also consider that, when you deal with a complex fistulising disease in the presence of a thickened mesentery (adenomesenteritis), intracorporeal ligation of vessels is technically demanding, and to extract the specimen or perform an extracorporeal anastomosis, a fairly large extraction site is often required.

The LIR!C study group has recently demonstrated that laparoscopic ileocecal resection is a safe alternative to infliximab in the case of a localised (<40 cm) inflammatory terminal ileitis after the failure of the conventional therapy [65]. This randomised, multicenter, controlled, open-label trial enrolled patients with nonstricturing ileocecal Crohn's disease, in whom glucocorticosteroids, thiopurines, or methotrexate had previously failed. The primary outcome was one-year quality of life based on the evaluation of the Inflammatory Bowel Disease Questionnaire (IBDQ), 12 months after treatment. Secondary outcomes took into account items like general quality of life, assessed with the Short Form-36 (SF-36), including its physical and mental domains, days of inability to participate in social life, and aesthetic results. In the LIR!C study, laparoscopic ileocecal resection, although not superior, appeared to be similar to infliximab treatment in terms of restoring the quality of life (mean IBDQ: 178 vs. 172 at 12 months) and is not associated with significantly increased morbidity. More, long-term follow-up reveals that more than one-third of patients in the infliximab group will require surgery anyway after 70 weeks, whereas only a quarter of patients who receive resection will need anti-TNF medical therapy within 112 weeks.

The topic of minimally invasive approach in abdominal surgery also requires emphasising what is now referred to as the “ERAS” protocol (Enhanced Recovery Programme after Surgery), which has been widely adopted also in the field of IBD and is made of several preoperative, intraoperative, and postoperative measures, to achieve a better and faster postoperative recovery.

This novel type of perioperative management was first described by Kehlet [66]. It was referred to as the “fast-track” protocol and initially applied to open surgery.

As the benefits coming from the application of the fast-track protocol were soon evident and appreciated, lots of researches have been carried out in this field and current programmes are usually very well structured and mainly called ERAS programmes. They include several elements, like no bowel preparation or fasting, preoperative carbohydrate load, no nasogastric tube, no abdominal drains, early removal of urinary catheter, early mobilisation and dietary intake, and early quitting of intravenous fluid, other than opioid-sparing analgesia. A case-matched study [67] focused on the application of the ERAS pathway after laparoscopic ileocecal resection for CD showed additional benefits in terms of faster recovery of bowel function and shorter hospital stay in the ERAS group over the conventional care-matched group.

It has been widely debated whether laparoscopy is the key factor to influence the results of ERAS programmes or is just one of the elements of ERAS itself. In other words, if we implement an ERAS protocol in open surgery, would we obtain the same results as in laparoscopy plus ERAS? The EnROL trial [68] tried to address this issue, concluding that even within an ERAS protocol, laparoscopic surgery gives an additional advantage in terms of length of hospital stay over open surgery.

## 5. Perianal Crohn's Disease and Rectovaginal Fistulas

Perianal fistulas in Crohn's disease were first described by Penner and Crohn in 1938 [69]. Perianal Crohn's disease encompasses a wide variety of entities, including both fistulising lesions (such as abscess, fistulas, or rectovaginal fistulas) and nonfistulising diseases (e.g., fissures, deep ulcers, anorectal strictures, skin tags, or haemorrhoids).

Moreover, the clinical impact of these entities can also vary significantly from asymptomatic and mild diseases to severe and devastating scenarios. While fissures, tags, or haemorrhoids would usually require only conservative management, abscesses and fistulas could require an aggressive medical and surgical intervention; that is why major attention to perianal Crohn's disease focuses on epidemiology, diagnosis, and management of abscesses and fistulas.

In population-based historical studies [5, 7, 70], the occurrence of perianal fistulas in Crohn's disease varies between 14 and 23%, with a cumulative incidence based on the time elapsed from the diagnosis. Perianal disease can also precede intestinal symptoms or appear at the time of diagnosis or even later during the follow-up. The risk of developing fistulising anal complications also depends on the disease site: colonic disease with rectal involvement is the most frequent association. The prevalence of the fistulising anal disease in these latter locations has been described as being up to 92%, while the prevalence was 41% for colonic disease with rectal sparing [6].

The first population-based study was led by Hellers et al. [6] who analysed the natural history of fistulising Crohn's disease among the residents of Stockholm County, Sweden, from 1955 to 1974; the cumulative incidence of anal fistula was 23%. After a mean follow-up of 9.4 years, 51% of the patients were healed, 9% had an open fistula, and 40% underwent proctectomy. These results could also reflect the natural history of perianal Crohn's disease before the wide spread of new treatments (e.g., immunomodulators and anti-TNF), which are likely to change the natural course of the disease.

Evidence from the Olmsted County cohort [5] showed that, at least, 1 perianal fistula occurred in 21% of patients, with a female-to-male ratio of 1.2 : 1, and this was the same ratio in the members of the cohort who did not develop fistulas. The cumulative risk of developing, at least, 1 perianal fistula was 12% after 1 year, 15% after 5 years, 21% after 10 years, and 26% after 20 years. Forty-four percent of these patients developed a fistula before or after the diagnosis of Crohn's disease, and similar data were reported from Stockholm County [6].

Perianal disease is generally associated with a more aggressive disease, and several factors have been studied to assess their potential influence on developing a perianal disease. For example, it has been shown that patients with a perianal disease are more likely to be steroid-resistant [71] as well as develop extraintestinal manifestations [72].

The natural history of fistulising perianal disease may also depend on the fistula's phenotype. Bell et al. [73] describe different courses in simple vs. complex fistulas in a referral centre; relapse occurred in 32% of simple fistulas and 23% of complex fistulas, with a mean relapse time of 30 and 11 months, respectively. Furthermore, complex fistulas required a median of six treatments to heal, while a median of three treatments was required in simple fistulas. Finally, the highest operative rate (including proctectomy, ostomy, or resection) was 50% in complex fistulas vs. only 6% in simple fistulas. A proctectomy rate of 50% in complex fistulas has been previously reported by Keighley and Allan [74] too.

The development of appropriate diagnostic tools is essential considering the incidence of the perianal CD, its prognostic implications, and a significant number of cases where the onset of perianal symptoms may precede the diagnosis.

The diagnostic algorithm of fistulising perianal CD includes three different imaging techniques as its focal points:

1. Examination under anaesthesia (EUA)
2. Contrast-enhanced pelvic magnetic resonance imaging (MRI)
3. Endoanal ultrasonography (EUS)

As a matter of fact, the importance of EUA is seen especially in the presence of sepsis. In this latter case, examination under anaesthesia, along with surgical drainage and/or seton placement, can be considered a first-line procedure to avoid any diagnostic delay related to MRI or EUS unavailability [75].

According to the latest ECCO CD guidelines [33], contrast-enhanced pelvic magnetic resonance imaging (MRI) is generally considered the initial procedure for the assessment of fistulising perianal CD (EL2). On the other hand, if rectal stenosis can be excluded, endoanal ultrasound is considered a valid alternative and equivalent to MRI when performed by expert hands. Examination under anaesthesia (EUA), finally, can be considered the gold standard for diagnosis, again, only if performed by experienced IBD surgeons. Even better, the sensitivity and specificity of both diagnostic modalities definitely get increased when combined with EUA.

There is no real consensus on a classification system for fistulising perianal CD. From a surgical point of view, Park et al.'s classification [76] is very useful, even in the decision-making process. On the other hand, some people classify perianal fistulas as just simple and complex ones.

In addition, Van Assche's severity score based on the MRI for perianal Crohn's disease [77] is a classification system worthy to be mentioned. It analyses several fistula features, thus evaluating CD severity (single/double tract, branching, location, extension, collections, and rectal wall involvement).

A specific sign of Crohn's ultrasound fistula (CUFS) was described by Zawadzki et al. [78] and further analysed by Zbar et al. [79]. It is defined as a hypoechogenic fistula tract or collection with a thin hypoechogenic rim and a surrounding hyperechoic region.

Surgical management of fistulising perianal CD mainly depends on the classification of perianal fistulas. In the presence of a perianal abscess, the procedure of choice is an urgent drainage. Once perianal sepsis gets controlled, simple asymptomatic fistulas do not usually require any further treatment [80]. Then, a “cone-like” fistulectomy with a draining seton is generally recommended in case of symptomatic fistulas or complex disease; the lay open technique is rarely suggested because of the disease's recurrent nature together with the consequent risk of incontinence.

The advent of biologics has radically changed the approach to fistulising perianal CD. In a randomised trial, infliximab turned out to be effective in inducing perianal fistula closure; moreover, it maintained the response for one year [81]. Similar results were evidenced in the ACCENT II trial, where 36% of patients treated with adalimumab had a complete closure compared to 19% of patients treated with placebo at week 54.

By considering the results obtained with biologics, current evidence suggests that the best way to deal with the complex fistulising perianal disease is a combined approach of both infliximab and seton placement [80]. Once the local sepsis with surgical drainage and seton has been controlled, the biologic therapy should begin. This is especially true when the anal fistula is associated with active proctitis; in this case, medical therapy is also useful to deal with luminal disease.

While infliximab has been shown to be effective in the process of fistula healing and closure, there is still some discussion about the timing for seton removal as the fistula will obviously not close until the seton is still in place. This is definitively a novelty in surgical management of perianal CD, since it is widely known that, once the local sepsis is under control, a loose seton has been considered a lifelong option to prevent recurrence in the CD.

Biologics have changed the dogma; then, some careful clinical evaluation is needed to establish the timing for seton removal together with the duration of medical therapy. Most authors would agree that, after surgical drainage and seton placement, biologic therapy should be initiated and a clinical reassessment made 6–8 weeks after the induction with infliximab. Only then may the seton removal be considered by keeping up with medical treatment as well as by making sure that the local control is achieved and there is no sepsis or active drainage or proctitis.

In regard to the “biosurgical” approach to the fistulising perianal CD, stem cells are a very promising tool. Panés et al. [82] recently published the results of a multicenter, randomised, double-blind, parallel-group, placebo-controlled study which involved 49 hospitals in seven European countries plus Israel. The study compared the use of a single local injection of expanded allogenic adipose-derived mesenchymal stem cells (Cx601) vs. placebo for complex perianal CD fistulas.

In this phase 3 study, 212 patients were randomly assigned to the stem cell group (107) or to the placebo group (105). Patients were eligible after the failure of, at least, one of the antibiotics, immunomodulators, or anti-TNF therapies.

The primary endpoint was the “combined remission at week 24,” which was meant as clinical remission (absence of discharge at delicate finger pressure) and radiological remission (absence of collections greater than 2 cm at MRI). After 24 weeks, a significantly high percentage of patients treated with Cx601 achieved the combined remission, 50% vs. 34% of the placebo group.

However, the difference in rates of clinical remission between the groups was not very significant. More encouraging results came from the favourable safety profile. Besides that, results of a longer follow-up were published [83]: at 52 weeks, they showed a higher proportion of combined (51.5% vs. 35.6%) and clinical remissions (59.2% vs. 41.6% of controls, for a difference of 17.6%, 95% CI, 4.1%–1.1%, *P* = 0.013). The safety profile was confirmed too.

With regard to surgical treatments, a rectal sleeve advancement flap [84] is sometimes an option for the primary closure of perianal fistulas in the case of a single internal opening and in the absence of rectal inflammation. Additionally, therapy with antibiotics or azathioprine should be considered a second-line therapy in case of failure of the combined approach. Unfortunately, proctectomy is still needed as the last resort chance in severe and complex fistulising anal disease.

The development of rectovaginal or pouch-vaginal fistulas is an even more challenging situation within the anal fistula CD topic.

Crohn's disease (CD) is the second most common cause of rectovaginal fistula (RVF) after obstetric lesions and has been reported to occur in approximately 10% of women with CD [85, 86]. There are a substantial lack of good evidence about the optimal treatment of rectovaginal fistulas and poor clear guidelines about the optimal therapeutic approach. Current knowledge is mainly based on the outcomes of case-series studies and some review articles [87–90], other than on expert opinions. The clinical classification of RVF is usually based on the relationship with the sphincter complex. If the internal opening is in the anal canal, they should be correlated as “anovaginal”; however, if the vaginal opening is very low, they are referred to as anovestibular or anointroital. When the opening is above the sphincter complex, they are correctly named as rectovaginal.

Low superficial anovaginal or anointroital fistulas might be laid opened or excised; on the other hand, this will likely create a deformity of the anal canal (keyhole deformity) with the risk of faecal soiling [91, 92]. This is the reason why the current management relies on local sepsis drainage and why surgical treatment is avoided if the fistula is asymptomatic.

Medical management of RVF is usually based on the use of antibiotics combined with metronidazole, which successfully treats some RVFs in association with surgical therapy [93, 94]; on the other hand, there are no randomised controlled data that support the use of antibiotics in the healing of Crohn's fistula.

The introduction of infliximab has been a major advancement in the treatment of fistulising CD. As part of the ACCENT II trial, Sands et al. [95] specifically examined the effect of infliximab on RVF related to CD. Twenty-five women with RVF were enrolled, and after induction, the first responders were randomised to continue receiving infliximab or placebo. They eventually concluded that infliximab was effective in the short-term closure of RVF (initial response rate was 64%) and maintenance therapy was more effective than placebo in prolonging closure.

Local RVF repair may be attempted when optimal local conditions exist. The presence of acute perianal sepsis or anorectal inflammation must be addressed before any repair attempts and a waiting period of 3–6 months may be necessary. Therefore, a temporary diverting stoma is required to achieve good control of local inflammation and infections in the great majority of cases. This has also been recently emphasised in a large series by Corte et al. [96] from Beaujon Hospital, reporting outcomes from 79 patients that underwent 286 procedures for rectovaginal fistulas; 34 patients (43%) had a CD-related RVF. The overall success rate at a median follow-up of 33 months was 72%; successful patients underwent a mean of 3.6 procedures each. Fifty-three percent of the procedures were performed in diverted patients; the presence of a stoma was associated with a better fistula healing rate, with the overall per-procedure success rate increasing from 6% (in nondiverted patients) to 32% (in procedures with stoma). They have also emphasised that the RVF healing rate is related to the type of procedure performed: even though a “step-up” surgical approach could be the rule for the majority of surgeons, they reported very low success rates for minor procedures such as seton drainage, fibrin glue or fistula plug, and advancement flaps (5%, 11%, and 12%, respectively). On the other hand, a higher per-procedure success rate has been reported for “major surgical procedures,” particularly gracilis muscle interposition (50%), biomesh interposition (44%), anterior resection with hand-sewn or stapled coloanal anastomosis (50%), and delayed coloanal anastomosis with transanal pull-through (91%). A per-procedure-based multivariate analysis showed how the following items are independent factors for success: major procedures (odds ratio (OR): 6.4 (2.9–14.2); *P* < 0.001), diverting stoma (OR: 3.5 (1.4–8.7); *P* = 0.009), less than nine months between diagnosis and the first surgery (OR: 2.3 (1.1–5.3); *P* = 0.046), and the first surgery in their institution (OR: 3.2 (1.5-6.9); *P* = 0.003). This latter aspect also underlines the importance of treating RVF in highly specialised referral centres. It should be noticed how CD is not found to be associated with poorer outcomes, and this is very likely due to authors' severe policy of not performing surgery on patients with active proctitis, eventually postposed after anti-TNF therapy.

Anyway, surgical repair can be subdivided into four main approaches: transanal, transvaginal, transperineal, or abdominal. The use of a loose seton in women with active anorectal disease can be prolonged indefinitely to preserve continence and delay or avoid a permanent stoma when local repair is not feasible [89, 93, 97, 98].

The anocutaneous flap is an option when low anovaginal fistulas are associated with anal stenosis; a flap made of perianal skin and subcutaneous tissue is mobilised into the anal canal. This can only be achieved if the perianal skin is soft and healthy, which is not common in Crohn's disease.

A 70% healing rate has been reported [99].

The rectal advancement flap repair is most suitable in patients with low fistulas, with no sphincter incontinence or muscle disruption, and with no significant rectal inflammation. The rectal advancement flap for rectovaginal fistula in CD was first described by Farkas and Gingold in 1983 [100]. A flap of the mucosa, submucosa, and rectal wall is created, starting only distally to the fistula opening, dissecting on the rectovaginal septum. Once sufficient mobilisation has been achieved, the fistula track is cored out and the flap is distally advanced, trimmed, and sutured up to the cut edge; the rectal opening is closed and covered by the flap while the vaginal orifice is left open for drainage.

This technique is generally addressed as the best treatment because the repair is performed from the high-pressure side of a high-pressure low shunt. Hull and Fazio [101] describe their experience in 35 patients who underwent three types of rectal flap repairs with a cumulative success rate of 67%; 50% of patients had a recurrence and 2 patients had a proctocolectomy and ileostomy. Kodner et al. [102] report 71% of primary healing after performing rectal advancement flap in 24 women with a spared rectum.

A “rectal sleeve advancement flap” has been described for the first time by Berman [103] and could be indicated when an extensive ulceration or stenosis of the anal canal prevents the possibility of fashioning a rectal advancement flap. It is based on performing a circumferential mucosectomy of the ulcerative mucosa and submucosa of the anal canal; rectal mobilisation is then continued until adequate mobility allows suturing the rectal flap without tension to the new dentate line. The fistula tract is always cored out, and the internal opening gets closed. The Cleveland Clinic [104] published their results with the rectal sleeve flap in 13 patients; 8 patients had proximal deviation, and a 60% success rate was achieved. The authors emphasised that this technique could be an alternative to proctectomy in carefully selected patients.

The vaginal advancement flap offers the advantage of using a healthy, flexible, and intact vaginal tissue to form the flap. Therefore, it represents a valid alternative in those situations showing a fibrotic and inflamed rectal mucosa where the rectal flap would be really difficult or impossible to perform [105]. Sher et al. [106] report their experience in 14 patients treated with vaginal flap; they achieved complete healing in 13 of them. The use of healthy vaginal tissue together with the levator ani muscle interposition is considered a key factor for their success rate.

Ruffolo et al. [90] led a systematic review to investigate the outcomes after the transrectal vs. transvaginal advancement flap. Eleven observational studies were eventually included with a total of 224 flap procedures for RVF in CD. The primary fistula closure pooled rate was 53.5% after the rectal flap and 61.1% after the vaginal flap. The overall fistula closure pooled rate was 74.6% after the rectal flap and 81% after the transvaginal approach. These differences were not significant; the authors conclude that the rectal flap should be considered the first choice, while the vaginal flap could be considered in case of anorectal or severe rectal stenosis.

Episioproctotomy is another technique to consider when an anterior sphincter defect coexists. A fistulectomy will be performed by creating a sort of fourth-degree perineal laceration; after the fistula is cored out and curetted, a sphincter overlap [107] is performed. The rectal mucosa is repaired, and the vaginal mucosa is approximated. A 71.4% healing rate was reported in a small series (8 women) [108].

The transverse perineal repair is based on dissecting the rectovaginal space through a transverse incision at the perineal body. After the scar and the fistula tract have been removed or curetted, the vaginal and rectal walls are closed in different layers and a levator plasty is performed. Athanasiadis et al. [109] in their comparative analysis of different techniques in a single institution series (56 procedures in 37 women) reported a success rate of 70% after transverse perineal repair, despite decreased postoperative resting pressures. The authors concluded that, as far as their experience is concerned, a higher success rate without significant impairment in continence is generally achieved with techniques that require a low degree of tissue mobilisation (direct closure or anocutaneous flap).

Tissue interposition techniques are aimed at interposing fresh, healthy, and well-vascularised tissue in the rectovaginal space, among the suture lines.

Gracilis muscle interposition has been proposed for Crohn's RVF, especially in case of failure after previous repairs. Wexner et al. [110] report their retrospective experience in 53 patients who underwent gracilis interposition; 15 had a rectovaginal fistula for CD and 2 had a pouch-vaginal fistula. Two patients required a second gracilis interposition. Only 33% of patients with CD-associated fistulas successfully healed compared to 75% of healing rate in non-CD patients. Furst et al. [111] performed the procedure in 12 women with recurrent RVF in CD; they obtained fistula healing in 11 patients; one rerecurrence was also documented. Zmora et al. [112] emphasise which factors are the key for a better outcome: faecal diversion and meticulous haemostasis together with a well-vascularised, tension-free muscle pedicle.

Another interposition technique is the use of the bulbocavernosus flap (Martius flap). Pitel et al. [113] report the largest series with Martius flap for RVF. Twenty patients underwent the procedure, with 8 patients (40%) presenting with a CD-related fistula. The overall success rate was 65%, while in CD patients, the healing rate was 50%.

Overall, there are too few studies on graciloplasty and Martius flap, and the limited number of patients makes definitive conclusions really difficult to draw.

The transperineal omenentum flap deserves to be cited among tissue interposition techniques. A left omental pedicle flap is created open or laparoscopically and is inserted deep in the rectovaginal space after an adequate mobilisation of the rectum and the vagina; the operation includes a perineal stage to treat the fistula and complete the reconstruction of the rectovaginal space. Schloericke et al. [114] report results of omentoplasty for RVF in the largest series published to date. They have treated 9 patients, but only one patient had a CD, and they obtained a success rate of 100% after a median follow-up of 22 months.

A new possibility for RVF treatment is represented by the use of a bioprosthetic fistula plug made of a lyophilised intestinal submucosa, which potentially allows the host cells to replace and repair the defective tissue. There are few reports describing the results, especially in the context of inflammatory bowel disease and RVF. Schwandner et al. [115] reported their experience in 21 patients with RVF; 9 had CD. After a median follow-up of 12 months, the success rate was 78% in patients with CD and 83% in those without CD after a primary mesh repair attempt. The authors suggest how the plus could also be used in combination with traditional advancement flap techniques. Gonsalves et al. [116] try to evaluate the short-term efficacy of the fistula plug in twelve patients with rectovaginal and pouch-vaginal fistula related to IBD. After a median follow-up of 15 months, 7 patients out of 12 (58%) were successfully treated after 20-plug insertions. The success rate after repeated plug insertions was 12.5%.

When most procedures are not feasible and the rectum is severely inflamed, one chance is given by the proctectomy with coloanal anastomosis; an immediate coloanal anastomosis can be performed or, alternatively, the option is the Turnbull-Cutait technique [117, 118]. The latter is based on an abdominoperineal endoanal pull-through resection, delaying the anastomosis from 5 to 7 days when local conditions are not satisfactory for an immediate anastomosis. El-Gazzaz et al. [108] report this technique in 7 patients with RVF and CD achieving a 57.1% healing rate.

Proctectomy is generally considered the last choice when other treatments have previously failed and local control cannot be achieved. On the other hand, proctectomy can also lead to major perineal complications, with high rates (20–50%) of chronic perineal sinus and delayed or even failed perineal wound healing [119, 120].

Few data are available regarding long-term outcomes and factors associated with failure in patients with RVF and CD. El-Gazzaz et al. [108] report outcomes in 75 women at a median follow-up of 44.6 months; different methods of repair were used, and the overall success rate was 46.2%. Smoking and steroid use were significantly associated with failure. Ruffolo et al. [121] analysed outcomes in 56 patients treated for RVF secondary to CD. Fistula closure has been achieved in 81% of patients, but often multiple operations have been required. Factors associated with failure were smoking, steroids, and previous extended colonic resection. On the other hand, infliximab was not associated with failure or delayed healing.

Pouch-vaginal fistulas (PVF) in CD is another challenging situation requiring surgeons' attention. Pouch surgery is generally not indicated in Crohn's disease due to its really high failure rate. Nevertheless, pouch-vaginal fistulas may occur often as a result of delayed CD diagnosis or when indeterminate colitis eventually evolves into Crohn's. There are no randomised controlled trials concerning PVF optimal management; the current knowledge lies on case-series studies, providing level IV evidence.

The overall incidence of PVF after ileoanal pouch anastomosis (IPAA) varies from 6 to 16%, with pouch failure occurring in 21 to 30% of these patients [122]. The late onset of PVF may be an indication of CD, especially in the absence of postoperative pelvic sepsis [123, 124].

A systematic review on surgical management and on factors associated with pouch-vaginal fistulas was led by Maslekar et al. [125]. Eighteen studies were finally included in the review. The authors emphasise how predisposing factors associated with PVF include technical factors (vaginal injuries or rectovaginal septum at the time of IPAA operation), septic factors (anastomotic leak, pelvic sepsis), and disease-related factors such as late diagnosis of Crohn's disease.

Basically, all the procedures described for RVF treatment have been adapted and used as options for PVF: seton drain, fistulectomy, transanal pouch advancement flap, transvaginal flap, tissue interposition techniques (Martius, gracilis, and omental flap), redo pouch, and biological plugs. Pouch excision is the last resort when all other treatments fail.

It is still a debated problem whether to divert and defunction before attempting PVF repair: a series of St Mark's Hospital reports have a 78.6% success rate in 14 patients who were all defunctioned [126]. Gorfine et al. [127] specifically examined the impact of faecal diversion on PVF repair and found no difference between defunctioned and nondefunctioned patients. A Cleveland Clinic study [128] retrospectively analysed the outcomes in 152 patients with pouch-vaginal fistulas. 77.3% of patients underwent local repair (ileal pouch advancement flap or transvaginal repair); redo ileal pouch was also performed in 19 patients. At the median follow-up of 83 months, pouch failure occurred overall in 35% of the patients. A multivariate analysis revealed that a current diagnosis of CD was the only independent risk factor for pouch failure: in CD patients, the healing of the fistula was significantly lower (22% vs. 73%) and the pouch failure significantly higher (52.7% vs. 22.7%).

## 6. Conclusions

Crohn's disease is a complex and heterogeneous disease, whose management requires a multidisciplinary approach, with close collaboration among surgeons, gastroenterologists, radiologists, and staff nurses.

Advances in surgical and medical therapy are changing the course of the disease. Nowadays, the introduction of both laparoscopy and novel surgical techniques, the improvement of recovery pathways, and the opening of new frontiers are allowing healthcare professionals to deal with complex and recurrent scenarios, trying to spare bowel and anal function, thus ensuring a better quality of life for the patient.

## References

[1] Gomollón F., Dignass A., Annese V. (2017). 3rd European evidence-based consensus on the diagnosis and management of Crohn’s disease 2016: part 1: diagnosis and medical management. *Journal of Crohn's & Colitis*.

[2] Henriksen M., Jahnsen J., Lygren I. (2007). Clinical course in Crohn’s disease: results of a five-year population-based follow-up study (the IBSEN study). *Scandinavian Journal of Gastroenterology*.

[3] Louis E., Collard A., Oger A. F., Degroote E., Aboul Nasr El Yafi F., Belaiche J. (2001). Behaviour of Crohn’s disease according to the Vienna classification: changing pattern over the course of the disease. *Gut*.

[4] Cosnes J., Cattan S., Blain A. (2002). Long-term evolution of disease behavior of Crohn’s disease. *Inflammatory Bowel Diseases*.

[5] Schwartz D. A., Loftus E. V., Tremaine W. J. (2002). The natural history of fistulizing Crohn’s disease in Olmsted County, Minnesota. *Gastroenterology*.

[6] Hellers G., Bergstrand O., Ewerth S., Holmstrom B. (1980). Occurrence and outcome after primary treatment of anal fistulae in Crohn’s disease. *Gut*.

[7] Tang L. Y., Rawsthorne P., Bernstein C. N. (2006). Are perineal and luminal fistulas associated in Crohn’s disease? A population-based study. *Clinical Gastroenterology and Hepatology*.

[8] Sandborn W. J., Hanauer S. B., Rutgeerts P. (2007). Adalimumab for maintenance treatment of Crohn’s disease: results of the CLASSIC II trial. *Gut*.

[9] Hanauer S. B., Sandborn W. J., Rutgeerts P. (2006). Human anti–tumor necrosis factor monoclonal antibody (adalimumab) in Crohn’s disease: the CLASSIC-I trial. *Gastroenterology*.

[10] Amezaga A. J., Van Assche G. (2016). Practical approaches to “top-down” therapies for Crohn’s disease. *Current Gastroenterology Reports*.

[11] Frolkis A. D., Dykeman J., Negrón M. E. (2013). Risk of surgery for inflammatory bowel diseases has decreased over time: a systematic review and meta-analysis of population-based studies. *Gastroenterology*.

[12] Hasegawa H., Watanabe M., Nishibori H., Okabayashi K., Hibi T., Kitajima M. (2003). Laparoscopic surgery for recurrent Crohn’s disease. *British Journal of Surgery*.

[13] Weston L. A., Roberts P. L., Schoetz D. J., Coller J. A., Murray J. J., Rusin L. C. (1996). Ileocolic resection for acute presentation of Crohn’s disease of the ileum. *Diseases of the Colon & Rectum*.

[14] Kim N. K., Senagore A. J., Luchtefeld M. A. (1997). Long-term outcome after ileocecal resection for Crohn’s disease. *The American Surgeon*.

[15] Krause U. (1971). Early or late operation in the treatment of Crohn’s disease. *Scandinavian Journal of Gastroenterology*.

[16] Latella G., Caprilli R., Travis S. (2011). In favour of early surgery in Crohn’s disease: a hypothesis to be tested. *Journal of Crohn's and Colitis*.

[17] Bemelman W. A., Allez M. (2014). The surgical intervention: earlier or never?. *Best Practice & Research Clinical Gastroenterology*.

[18] Rispo A., Imperatore N., Testa A. (2018). Combined endoscopic/sonographic-based risk matrix model for predicting one-year risk of surgery: a prospective observational study of a tertiary centre severe/refractory Crohn’s disease cohort. *Journal of Crohn's and Colitis*.

[19] Fazio V. W., Marchetti F., Church J. M. (1996). Effect of resection margins on the recurrence of Crohn’s disease in the small bowel: a randomized controlled trial. *Annals of Surgery*.

[20] Heuman R., Boeryd B., Bolin T., Sjodahl R. (1983). The influence of disease at the margin of resection on the outcome of Crohn’s disease. *British Journal of Surgery*.

[21] Bemelman W. A., Warusavitarne J., Sampietro G. M. (2018). ECCO-ESCP consensus on surgery for Crohn’s disease. *Journal of Crohn's and Colitis*.

[22] Gervais D. A., Hahn P. F., O’Neill M. J., Mueller P. R. (2002). Percutaneous abscess drainage in Crohn disease: technical success and short- and long-term outcomes during 14 years. *Radiology*.

[23] Garcia J. C., Persky S. E., Bonis P. A. L., Topazian M. (2001). Abscesses in Crohn’s disease: outcome of medical versus surgical treatment. *Journal of Clinical Gastroenterology*.

[24] Zerbib P., Koriche D., Truant S. (2010). Pre-operative management is associated with low rate of post-operative morbidity in penetrating Crohn’s disease. *Alimentary Pharmacology & Therapeutics*.

[25] Aberra F. N., Lewis J. D., Hass D., Rombeau J. L., Osborne B., Lichtenstein G. R. (2003). Corticosteroids and immunomodulators: postoperative infectious complication risk in inflammatory bowel disease patients. *Gastroenterology*.

[26] Fumery M., Seksik P., Auzolle C. (2017). Postoperative complications after ileocecal resection in Crohn’s disease: a prospective study from the REMIND group. *The American Journal of Gastroenterology*.

[27] Colombel J. F., Loftus E. V., Tremaine W. J. (2004). Early postoperative complications are not increased in patients with Crohn’s disease treated perioperatively with infliximab or immunosuppressive therapy. *The American Journal of Gastroenterology*.

[28] Kunitake H., Hodin R., Shellito P. C., Sands B. E., Korzenik J., Bordeianou L. (2008). Perioperative treatment with infliximab in patients with Crohn’s disease and ulcerative colitis is not associated with an increased rate of postoperative complications. *Journal of Gastrointestinal Surgery*.

[29] Schluender S. J., Ippoliti A., Dubinsky M. (2007). Does infliximab influence surgical morbidity of ileal pouch-anal anastomosis in patients with ulcerative colitis?. *Diseases of the Colon & Rectum*.

[30] Brouquet A., Maggiori L., Zerbib P. (2018). Anti-TNF therapy is associated with an increased risk of postoperative morbidity after surgery for ileocolonic Crohn disease: results of a prospective nationwide cohort. *Annals of Surgery*.

[31] Yang Z., Wu Q., Wu K., Fan D. (2010). Meta-analysis: pre-operative infliximab treatment and short-term post-operative complications in patients with ulcerative colitis. *Alimentary Pharmacology & Therapeutics*.

[32] Appau K. A., Fazio V. W., Shen B. (2008). Use of infliximab within 3 months of ileocolonic resection is associated with adverse postoperative outcomes in Crohn’s patients. *Journal of Gastrointestinal Surgery*.

[33] Van Assche G., Dignass A., Reinisch W. (2010). The second European evidence-based consensus on the diagnosis and management of Crohn’s disease: special situations. *Journal of Crohn's and Colitis*.

[34] Auzolle C., Nancey S., Tran-Minh M. L. (2018). Male gender, active smoking and previous intestinal resection are risk factors for post-operative endoscopic recurrence in Crohn’s disease: results from a prospective cohort study. *Alimentary Pharmacology & Therapeutics*.

[35] Nunes T., Etchevers M. J., García-Sánchez V. (2016). Impact of smoking cessation on the clinical course of Crohn’s disease under current therapeutic algorithms: a multicenter prospective study. *The American Journal of Gastroenterology*.

[36] Simillis C., Purkayastha S., Yamamoto T., Strong S. A., Darzi A. W., Tekkis P. P. (2007). A meta-analysis comparing conventional end-to-end anastomosis vs. other anastomotic configurations after resection in Crohn’s disease. *Diseases of the Colon & Rectum*.

[37] He X., Chen Z., Huang J. (2014). Stapled side-to-side anastomosis might be better than handsewn end-to-end anastomosis in ileocolic resection for Crohn’s disease: a meta-analysis. *Digestive Diseases and Sciences*.

[38] Scarpa M., Angriman I., Barollo M. (2004). Role of stapled and hand-sewn anastomoses in recurrence of Crohn’s disease. *Hepato-Gastroenterology*.

[39] Kono T., Ashida T., Ebisawa Y. (2011). A new antimesenteric functional end-to-end handsewn anastomosis: surgical prevention of anastomotic recurrence in Crohn’s disease. *Diseases of the Colon & Rectum*.

[40] Anthony A., Dhillon A. P., Pounder R. E., Wakefield A. J. (1997). Ulceration of the ileum in Crohn’s disease: correlation with vascular anatomy. *Journal of Clinical Pathology*.

[41] Kono T., Fichera A., Maeda K. (2016). Kono-S anastomosis for surgical prophylaxis of anastomotic recurrence in Crohn’s disease: an international multicenter study. *Journal of Gastrointestinal Surgery*.

[42] Luglio G., Rispo A., Castiglione F. (2016). Kono-type anastomosis in a patient with severe multi-recurrent Crohn’s disease. *International Journal of Colorectal Disease*.

[43] Luglio G., Rispo A., Peltrini R. (2016). P587. SuPREMe-CD Study: surgical prevention of anastomotic recurrence by excluding mesentery in Crohn’s disease—preliminary results and trial protocol. *Journal of Crohn's and Colitis*.

[44] Coffey C. J., Kiernan M. G., Sahebally S. M. (2018). Inclusion of the mesentery in ileocolic resection for Crohn’s disease is associated with reduced surgical recurrence. *Journal of Crohn's and Colitis*.

[45] Alexander-Williams J. (1986). The technique of intestinal strictureplasty. *International Journal of Colorectal Disease*.

[46] Hurst R. D., Michelassi F. (1998). Strictureplasty for Crohn’s disease: techniques and long-term results. *World Journal of Surgery*.

[47] Michelassi F. (1996). Side-to-side isoperistaltic strictureplasty for multiple Crohn’s strictures. *Diseases of the Colon & Rectum*.

[48] Michelassi F., Upadhyay G. A. (2004). Side-to-side isoperistaltic strictureplasty in the treatment of extensive Crohn’s disease. *The Journal of Surgical Research*.

[49] Tonelli F., Fedi M., Paroli M. G., Fazi M. (2004). Indications and results of side-to-side isoperistaltic strictureplasty in Crohn’s disease. *Diseases of the Colon & Rectum*.

[50] Tichansky D., Cagir B., Yoo E., Marcus S. M., Fry R. D. (2000). Strictureplasty for Crohn’s disease: meta-analysis. *Diseases of the Colon & Rectum*.

[51] Michelassi F., Taschieri A., Tonelli F. (2007). An international, multicenter, prospective, observational study of the side-to-side isoperistaltic strictureplasty in Crohn’s disease. *Diseases of the Colon & Rectum*.

[52] Yamamoto T., Fazio V. W., Tekkis P. P. (2007). Safety and efficacy of strictureplasty for Crohn’s disease: a systematic review and meta-analysis. *Diseases of the Colon & Rectum*.

[53] Fearnhead N. S., Chowdhury R., Box B., George B. D., Jewell D. P., Mortensen N. J. M. C. (2006). Long-term follow-up of strictureplasty for Crohn’s disease. *British Journal of Surgery*.

[54] Menon A. M., Mirza A. H., Moolla S., Morton D. G. (2007). Adenocarcinoma of the small bowel arising from a previous strictureplasty for Crohn’s disease: report of a case. *Diseases of the Colon & Rectum*.

[55] Luglio G., De Palma G. D., Tarquini R. (2015). Laparoscopic colorectal surgery in learning curve: role of implementation of a standardized technique and recovery protocol. A cohort study. *Annals of Medicine and Surgery*.

[56] Luglio G., Nelson H. (2010). Laparoscopy for colon cancer: state of the art. *Surgical Oncology Clinics of North America*.

[57] West N. P., Kennedy R. H., Magro T. (2014). Morphometric analysis and lymph node yield in laparoscopic complete mesocolic excision performed by supervised trainees. *British Journal of Surgery*.

[58] Milone M., Elmore U., Di Salvo E. (2015). Intracorporeal versus extracorporeal anastomosis. Results from a multicentre comparative study on 512 right-sided colorectal cancers. *Surgical Endoscopy*.

[59] Milson J. W., Hammerhofer K. A., Böhm B., Marcello P., Elson P., Fazio V. W. (2001). Prospective, randomized trial comparing laparoscopic vs. conventional surgery for refractory ileocolic Crohn’s disease. *Diseases of the Colon & Rectum*.

[60] Maartense S., Dunker M. S., Slors J. F. M. (2006). Laparoscopic-assisted versus open ileocolic resection for Crohn’s disease: a randomized trial. *Annals of Surgery*.

[61] Eshuis E. J., Polle S. W., Slors J. F. (2008). Long-term surgical recurrence, morbidity, quality of life, and body image of laparoscopic-assisted vs. open ileocolic resection for Crohn’s disease: a comparative study. *Diseases of the Colon & Rectum*.

[62] Goyer P., Alves A., Bretagnol F., Bouhnik Y., Valleur P., Panis Y. (2009). Impact of complex Crohn’s disease on the outcome of laparoscopic ileocecal resection: a comparative clinical study in 124 patients. *Diseases of the Colon & Rectum*.

[63] Alves A., Panis Y., Bouhnik Y. (2005). Factors that predict conversion in 69 consecutive patients undergoing laparoscopic ileocecal resection for Crohn’s disease: a prospective study. *Diseases of the Colon & Rectum*.

[64] Giglio M. C., Celentano V., Tarquini R., Luglio G., De Palma G. D., Bucci L. (2015). Conversion during laparoscopic colorectal resections: a complication or a drawback? A systematic review and meta-analysis of short-term outcomes. *International Journal of Colorectal Disease*.

[65] Ponsioen C. Y., de Groof E. J., Eshuis E. J. (2017). Laparoscopic ileocaecal resection versus infliximab for terminal ileitis in Crohn’s disease: a randomised controlled, open-label, multicentre trial. *The Lancet Gastroenterology & Hepatology*.

[66] Kehlet H. (1997). Multimodal approach to control postoperative pathophysiology and rehabilitation. *British Journal of Anaesthesia*.

[67] Spinelli A., Bazzi P., Sacchi M. (2013). Short-term outcomes of laparoscopy combined with enhanced recovery pathway after ileocecal resection for Crohn’s disease: a case-matched analysis. *Journal of Gastrointestinal Surgery*.

[68] Kennedy R. H., Francis E. A., Wharton R. (2014). Multicenter randomized controlled trial of conventional versus laparoscopic surgery for colorectal cancer within an enhanced recovery programme: EnROL. *Journal of Clinical Oncology*.

[69] Penner A., Crohn B. B. (1938). Perianal fistulae as a complication of regional ileitis. *Annals of Surgery*.

[70] Lapidus A. (2006). Crohn’s disease in Stockholm County during 1990-2001: an epidemiological update. *World Journal of Gastroenterology*.

[71] Gelbmann C. M., Rogler G., Gross V. (2002). Prior bowel resections, perianal disease, and a high initial Crohn’s disease activity index are associated with corticosteroid resistance in active Crohn’s disease. *The American Journal of Gastroenterology*.

[72] Rankin G. B., Watts H. D., Melnyk C. S., Kelley M. L. (1979). National Cooperative Crohn’s Disease Study: extraintestinal manifestations and perianal complications. *Gastroenterology*.

[73] Bell S. J., Williams A. B., Wiesel P., Wilkinson K., Cohen R. C. G., Kamm M. A. (2003). The clinical course of fistulating Crohn’s disease. *Alimentary Pharmacology & Therapeutics*.

[74] Keighley M. R. B., Allan R. N. (1986). Current status and influence of operation on perianal Crohn’s disease. *International Journal of Colorectal Disease*.

[75] Gecse K. B., Bemelman W., Kamm M. A. (2014). A global consensus on the classification, diagnosis and multidisciplinary treatment of perianal fistulising Crohn’s disease. *Gut*.

[76] Parks A. G., Gordon P. H., Hardcastle J. D. (1976). A classification of fistula-in-ano. *British Journal of Surgery*.

[77] Kulkarni S., Gomara R., Reeves-Garcia J., Hernandez E., Restrepo R. (2014). MRI-based score helps in assessing the severity and in follow-up of pediatric patients with perianal Crohn disease. *Journal of Pediatric Gastroenterology and Nutrition*.

[78] Zawadzki A., Starck M., Bohe M., Thorlacius H. (2012). A unique 3D endoanal ultrasound feature of perianal Crohn’s fistula: the ‘Crohn ultrasound fistula sign’. *Colorectal Disease*.

[79] Zbar A. P., Horesh N., Bucholtz V., Zmora O., Beer-Gabel M., Carter D. (2013). Are there specific endosonographic features in Crohn’s patients with perianal fistulae?. *Journal of Crohn's & Colitis*.

[80] Orlando A., Armuzzi A., Papi C. (2011). The Italian Society of Gastroenterology (SIGE) and the Italian Group for the Study of Inflammatory Bowel Disease (IG-IBD) clinical practice guidelines: the use of tumor necrosis factor-alpha antagonist therapy in inflammatory bowel disease. *Digestive and Liver Disease*.

[81] Present D. H., Rutgeerts P., Targan S. (1999). Infliximab for the treatment of fistulas in patients with Crohn’s disease. *The New England Journal of Medicine*.

[82] Panés J., García-Olmo D., van Assche G. (2016). Expanded allogeneic adipose-derived mesenchymal stem cells (Cx601) for complex perianal fistulas in Crohn’s disease: a phase 3 randomised, double-blind controlled trial. *The Lancet*.

[83] Panés J., García-Olmo D., van Assche G. (2018). Long-term efficacy and safety of stem cell therapy (Cx601) for complex perianal fistulas in patients with Crohn’s disease. *Gastroenterology*.

[84] Soltani A., Kaiser A. M. (2010). Endorectal advancement flap for cryptoglandular or Crohn’s fistula-in-ano. *Diseases of the Colon & Rectum*.

[85] Radcliffe A. G., Ritchie J. K., Hawley P. R., Lennard-Jones J. E., Northover J. M. A. (1988). Anovaginal and rectovaginal fistulas in Crohn’s disease. *Diseases of the Colon & Rectum*.

[86] Saclarides T. J. (2002). Rectovaginal fistula. *The Surgical Clinics of North America*.

[87] Valente M. A., Hull T. L. (2014). Contemporary surgical management of rectovaginal fistula in Crohn’s disease. *World Journal of Gastrointestinal Pathophysiology*.

[88] Zhu Y. F., Tao G. Q., Zhou N., Xiang C. (2011). Current treatment of rectovaginal fistula in Crohn’s disease. *World Journal of Gastroenterology*.

[89] Andreani S. M., Dang H. H., Grondona P., Khan A. Z., Edwards D. P. (2007). Rectovaginal fistula in Crohn’s disease. *Diseases of the Colon & Rectum*.

[90] Ruffolo C., Scarpa M., Bassi N., Angriman I. (2010). A systematic review on advancement flaps for rectovaginal fistula in Crohn’s disease: transrectal vs transvaginal approach. *Colorectal Disease*.

[91] Scott N. A., Nair A., Hughes L. E. (1992). Anovaginal and rectovaginal fistula in patients with Crohn’s disease. *British Journal of Surgery*.

[92] Levy C., Tremaine W. J. (2002). Management of internal fistulas in Crohn’s disease. *Inflammatory Bowel Diseases*.

[93] Hannaway C. D., Hull T. L. (2008). Current considerations in the management of rectovaginal fistula from Crohn’s disease. *Colorectal Disease*.

[94] Bernstein L. H., Frank M. S., Brandt L. J., Boley S. J. (1980). Healing of perineal Crohn’s disease with metronidazole. *Gastroenterology*.

[95] Sands B. E., Blank M. A., Patel K., van Deventer S., ACCENT II Study (2004). Long-term treatment of rectovaginal fistulas in Crohn’s disease: response to infliximab in the ACCENT II Study. *Clinical Gastroenterology and Hepatology*.

[96] Corte H., Maggiori L., Treton X., Lefevre J. H., Ferron M., Panis Y. (2015). Rectovaginal fistula. *Annals of Surgery*.

[97] Faucheron J. L., Saint-Marc O., Guibert L., Parc R. (1996). Long-term seton drainage for high anal fistulas in Crohn’s disease--a sphincter-saving operation?. *Diseases of the Colon & Rectum*.

[98] Thornton M., Solomon M. J. (2005). Long-term indwelling seton for complex anal fistulas in Crohn’s disease. *Diseases of the Colon & Rectum*.

[99] Hesterberg R., Schmidt W. U., Müller F., Röher H. D. (1993). Treatment of anovaginal fistulas with an anocutaneous flap in patients with Crohn’s disease. *International Journal of Colorectal Disease*.

[100] Farkas A. M., Gingold B. S. (1983). Repair of rectovaginal fistula in Crohn’s disease by rectal mucosal advancement flap. *Mount Sinai Journal of Medicine*.

[101] Hull T. L., Fazio V. W. (1997). Surgical approaches to low anovaginal fistula in Crohn’s disease. *American Journal of Surgery*.

[102] Kodner I. J., Mazor A., Shemesh E. I., Fry R. D., Fleshman J. W., Birnbaum E. H. (1993). Endorectal advancement flap repair of rectovaginal and other complicated anorectal fistulas. *Surgery*.

[103] Berman I. R. (1991). Sleeve advancement anorectoplasty for complicated anorectal/vaginal fistula. *Diseases of the Colon & Rectum*.

[104] Marchesa P., Hull T. L., Fazio V. W. (1998). Advancement sleeve flaps for treatment of severe perianal Crohn’s disease. *British Journal of Surgery*.

[105] Penninckx F., Moneghini D., D'Hoore A., Wyndaele J., Coremans G., Rutgeerts P. (2001). Success and failure after repair of rectovaginal fistula in Crohn’s disease: analysis of prognostic factors. *Colorectal Disease*.

[106] Sher M. E., Bauer J. J., Gelernt I. (1991). Surgical repair of rectovaginal fistulas in patients with Crohn’s disease: transvaginal approach. *Diseases of the Colon & Rectum*.

[107] Hull T. L., El-Gazzaz G., Gurland B., Church J., Zutshi M. (2011). Surgeons should not hesitate to perform episioproctotomy for rectovaginal fistula secondary to cryptoglandular or obstetrical origin. *Diseases of the Colon & Rectum*.

[108] El-Gazzaz G., Hull T., Mignanelli E., Hammel J., Gurland B., Zutshi M. (2010). Analysis of function and predictors of failure in women undergoing repair of Crohn’s related rectovaginal fistula. *Journal of Gastrointestinal Surgery*.

[109] Athanasiadis S., Yazigi R., Kohler A., Helmes C. (2007). Recovery rates and functional results after repair for rectovaginal fistula in Crohn’s disease: a comparison of different techniques. *International Journal of Colorectal Disease*.

[110] Wexner S. D., Ruiz D. E., Genua J., Nogueras J. J., Weiss E. G., Zmora O. (2008). Gracilis muscle interposition for the treatment of rectourethral, rectovaginal, and pouch-vaginal fistulas: results in 53 patients. *Annals of Surgery*.

[111] Furst A., Schmidbauer C., Swol-Ben J., Iesalnieks I., Schwandner O., Agha A. (2008). Gracilis transposition for repair of recurrent anovaginal and rectovaginal fistulas in Crohn’s disease. *International Journal of Colorectal Disease*.

[112] Zmora O., Tulchinsky H., Gur E., Goldman G., Klausner J. M., Rabau M. (2006). Gracilis muscle transposition for fistulas between the rectum and urethra or vagina. *Diseases of the Colon & Rectum*.

[113] Pitel S., Lefevre J. H., Parc Y., Chafai N., Shields C., Tiret E. (2011). Martius advancement flap for low rectovaginal fistula: short- and long-term results. *Colorectal Disease*.

[114] Schloericke E., Hoffmann M., Zimmermann M. (2012). Transperineal omentum flap for the anatomic reconstruction of the rectovaginal space in the therapy of rectovaginal fistulas. *Colorectal Disease*.

[115] Schwandner O., Fuerst A., Kunstreich K., Scherer R. (2009). Innovative technique for the closure of rectovaginal fistula using Surgisis mesh. *Techniques in Coloproctology*.

[116] Gonsalves S., Sagar P., Lengyel J., Morrison C., Dunham R. (2009). Assessment of the efficacy of the rectovaginal button fistula plug for the treatment of ileal pouch-vaginal and rectovaginal fistulas. *Diseases of the Colon & Rectum*.

[117] Kirwan W. O., Turnbull R. B., Fazio V. W., Weakley F. L. (1978). Pullthrough operation with delayed anastomosis for rectal cancer. *British Journal of Surgery*.

[118] Cutait D. E., Cutait R., Ioshimoto M., da Silva J. H., Manzione A. (1985). Abdominoperineal endoanal pull-through resection. A comparative study between immediate and delayed colorectal anastomosis. *Diseases of the Colon & Rectum*.

[119] Heyen F., Winslet M. C., Andrews H., Alexander-Williams J., Keighley M. R. B. (1989). Vaginal fistulas in Crohn’s disease. *Diseases of the Colon & Rectum*.

[120] Cohen J. L., Stricker J. W., Schoetz D. J., Coller J. A., Veidenheimer M. C. (1989). Rectovaginal fistula in Crohn’s disease. *Diseases of the Colon & Rectum*.

[121] Ruffolo C., Penninckx F., van Assche G. (2009). Outcome of surgery for rectovaginal fistula due to Crohn’s disease. *British Journal of Surgery*.

[122] Lolohea S., Lynch A. C., Robertson G. B., Frizelle F. A. (2005). Ileal pouch-anal anastomosis-vaginal fistula: a review. *Diseases of the Colon & Rectum*.

[123] Melton G. B., Fazio V. W., Kiran R. P. (2008). Long-term outcomes with ileal pouch-anal anastomosis and Crohn’s disease: pouch retention and implications of delayed diagnosis. *Annals of Surgery*.

[124] Tsujinaka S., Ruiz D., Wexner S. D. (2006). Surgical management of pouch-vaginal fistula after restorative proctocolectomy. *Journal of the American College of Surgeons*.

[125] Maslekar S., Sagar P. M., Harji D., Bruce C., Griffiths B. (2012). The challenge of pouch-vaginal fistulas: a systematic review. *Techniques in Coloproctology*.

[126] Burke D., van Laarhoven C. J. H. M., Herbst F., Nicholls R. J. (2001). Transvaginal repair of pouch-vaginal fistula. *British Journal of Surgery*.

[127] Gorfine S. R., Fichera A., Harris M. T., Bauer J. J. (2003). Long-term results of salvage surgery for septic complications after restorative proctocolectomy: does fecal diversion improve outcome?. *Diseases of the Colon & Rectum*.

[128] Mallick I. H., Hull T. L., Remzi F. H., Kiran R. P. (2014). Management and outcome of pouch-vaginal fistulas after IPAA surgery. *Diseases of the Colon & Rectum*.

